# Use of 3-dimensional printing at the point-of-care to manage a complex wound in hemifacial necrotizing fasciitis: a case report

**DOI:** 10.1186/s41205-022-00166-4

**Published:** 2023-02-23

**Authors:** Sarah C. Nyirjesy, Ryan T. Judd, Yazen Alfayez, Peter Lancione, Brian Swendseid, Natalia von Windheim, Stephen Nogan, Nolan B. Seim, Kyle K. VanKoevering

**Affiliations:** Department of Otolaryngology- Head and Neck Surgery, The James Cancer Hospital and Solove Research Institute, The Ohio State University Wexner Medical Center, 915 Olentangy River Road, Columbus, OH 43210 USA

**Keywords:** Three-dimensional printing (3D), Negative pressure wound therapy, Wound Vac, Computer assisted design, Necrotizing fasciitis, Case report, Expanded access for medical devices emergency use mechanism, Point of care

## Abstract

**Background:**

Complex facial wounds can be difficult to stabilize due to proximity of vital structures. We present a case in which a patient-specific wound splint was manufactured using computer assisted design and three-dimensional printing at the point-of-care to allow for wound stabilization in the setting of hemifacial necrotizing fasciitis. We also describe the process and implementation of the United States Food and Drug Administration Expanded Access for Medical Devices Emergency Use mechanism.

**Case presentation:**

A 58-year-old female presented with necrotizing fasciitis of the neck and hemiface. After multiple debridements, she remained critically ill with poor vascularity of tissue in the wound bed and no evidence of healthy granulation tissue and concern for additional breakdown towards the right orbit, mediastinum, and pretracheal soft tissues, precluding tracheostomy placement despite prolonged intubation. A negative pressure wound vacuum was considered for improved healing, but proximity to the eye raised concern for vision loss due to traction injury. As a solution, under the Food and Drug Administration’s Expanded Access for Medical Devices Emergency Use mechanism, we designed a three-dimensional printed, patient-specific silicone wound splint from a CT scan, allowing the wound vacuum to be secured to the splint rather than the eyelid.

After 5 days of splint-assisted vacuum therapy, the wound bed stabilized with no residual purulence and developed healthy granulation tissue, without injury to the eye or lower lid. With continued vacuum therapy, the wound contracted to allow for safe tracheostomy placement, ventilator liberation, oral intake, and hemifacial reconstruction with a myofascial pectoralis muscle flap and a paramedian forehead flap 1 month later. She was eventually decannulated and at six-month follow-up has excellent wound healing and periorbital function.

**Conclusions:**

Patient-specific, three-dimensional printing is an innovative solution that can facilitate safe placement of negative pressure wound therapy adjacent to delicate structures. This report also demonstrates feasibility of point-of-care manufacturing of customized devices for optimizing complex wound management in the head and neck, and describes successful use of the United States Food and Drug Administration’s Expanded Access for Medical Devices Emergency Use mechanism.

## Background

Necrotizing fasciitis (NF) is an uncommon, rapidly progressive infection of the subcutaneous tissue and superficial fascia with secondary necrosis of the overlying skin [1]. The disease is treated with a combination of intravenous antibiotics and aggressive surgical debridement to expose healthy, bleeding tissue [1, 2]. Most patients require multiple debridements, which can result in massive defects requiring reconstruction. NF rarely occurs in the head and neck [3]. In such cases, aggressive surgical debridement is still the standard of care, but is complicated by the proximity of infection to vital structures [2, 3]. Between and following debridements, NF in the head and neck, as in other sites, is typically managed with either wet to dry dressings [3–5] or negative pressure wound therapy (NPWT) via wound vacuum (wound vac) [6–8] until the wound bed is considered sufficiently well-healed for reconstruction. NPWT has been shown to have improved outcomes in certain infectious settings [3, 6, 7], and inability to use wound vac is associated with prolonged wound care, as well as significant pain from daily or even more frequent dressing changes [9, 10]. Difficulty in application of NPWT to complex facial wounds as well as the possibility of damage to the facial structures from negative pressure have limited its application to small case series in the head and neck [3, 6, 8, 9]. In the setting of complicated defects, point-of-care designed custom devices may be considered to maximize the preservation of surrounding vital structures while also promoting maximal wound healing.

We present the case of a massive hemifacial and neck wound from NF, for which a novel patient-specific, three-dimensional (3D) printed wound splint was manufactured at the point-of-care and placed under the Food and Drug Administration’s (FDA) Expanded Access for Medical Devices Emergency Use mechanism (EAEU), in order to prevent negative pressure on the ocular structures that allowed for adequate wound healing and satisfactory visual and reconstructive outcomes.

## Case presentation

A 58-year-old female presented with right facial and neck swelling and respiratory distress after a recent dental infection treated with clindamycin. On examination, there were grossly decayed teeth with a firm floor of mouth and diffuse erythema of the right neck, lower and mid-face. She was diagnosed with Ludwig’s Angina and immediately intubated due to stridor and pending airway compromise. Imaging revealed extensive infection and abscess formation involving the submandibular, pterygomandibular, and buccal spaces, and she was taken to the operating room for dental extraction and abscess drainage by the oral surgery team and started on broad spectrum antibiotics. Cultures from this operation were polymicrobial and consistent with oral microbes. On postoperative day 1, she developed rapidly expanding erythema and swelling of the right hemiface and neck with gross necrosis of the skin throughout the right neck and face. The Otolaryngology team was consulted for possible necrotizing fasciitis.

**Fig. 1:**
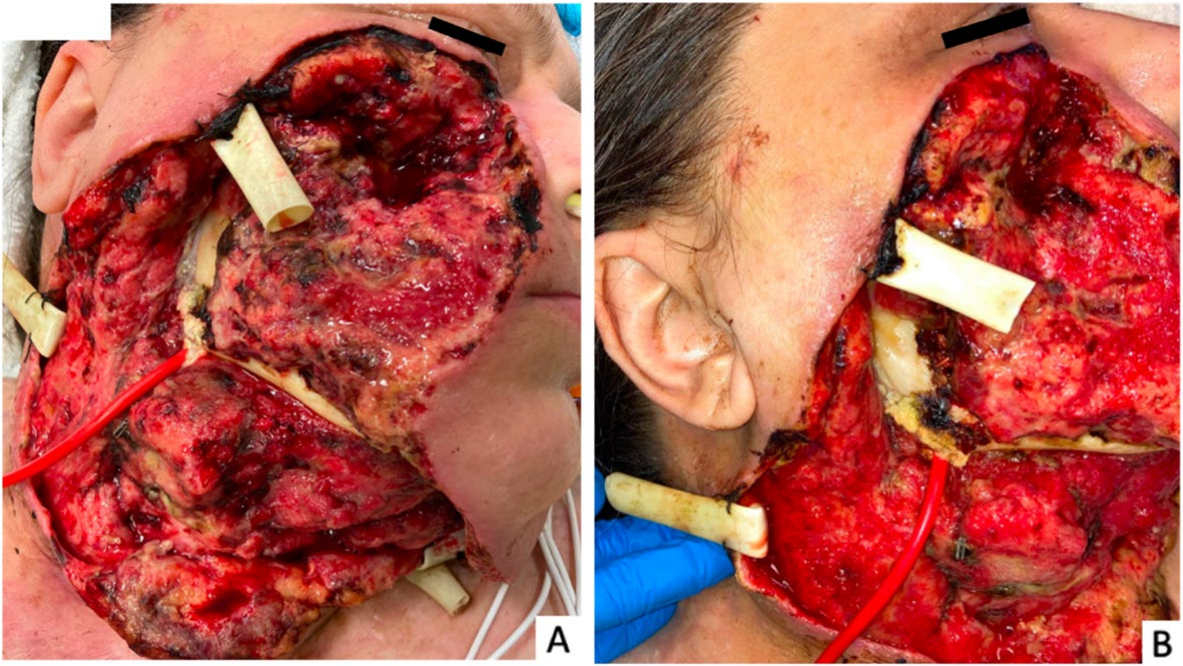
**a**, **b**) 58 year old female with right hemifacial wound from necrotizing fasciitis. Penrose drains and red rubber catheter in place for irrigations

The patient was taken to the operating room for urgent debridement, which included the skin of the right neck and face up to lower eyelid, devascularized orbicularis oculi, mylohyoid, superficial muscular aponeurotic system in the midface, masseter, and all branches of the facial nerve. The debridement ultimately required removal of a 20x30cm segment of the right and central neck and a 20x20cm segment of the right face (Fig. 1).

Subsequent debridement 2 days later required excision of soft tissue to the level of the zygoma and infraorbital rim. Penrose drains were placed to assist with drainage and irrigation of deeper spaces communicating with the open wound, including the pretracheal space. Antibiotics were transitioned to vancomycin, ampicillin-sulbactam, and fluconazole for a planned duration of 4 weeks, as was appropriate per bacterial culture susceptibilities.

The decision was made to proceed with twice daily local wound care with Dakins irrigations through the Penrose drains into the deep spaces, and placement of wet-to-dry Dakins-soaked gauze over exposed soft tissue between operative debridements. Ophthalmology was consulted for assistance with progressive ocular and lower eyelid involvement. Due to the extent of the wound, heavy sedation was required for dressing changes. However, due to open wound without overlying skin in the pretracheal space, a tracheostomy was not offered, as secretions would have saturated the wound bed and prolonged infection, resulting in a prolonged endotracheal intubation. Despite aggressive wound care, 12 days after initial aggressive debridement, purulent drainage continued to be encountered with irrigations, with concern for spread of infection threatening the right orbit and mediastinum. Furthermore, new areas of devitalized tissue were encountered routinely throughout the wound bed and debrided at bedside. The white blood cell count was persistently elevated throughout this time and the patient remained septic.

**Fig. 2:**
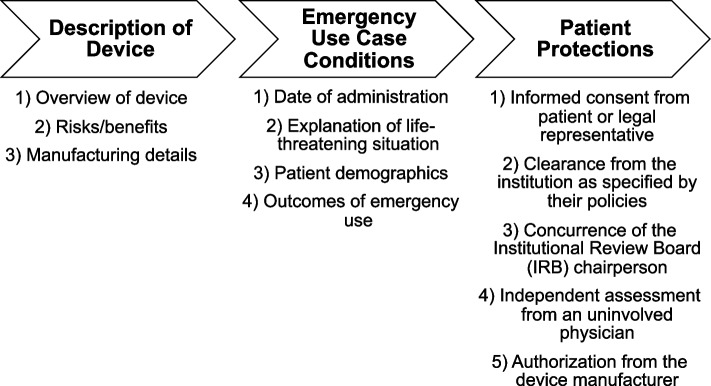
Process and documentation required for device placement under the United States Food and Drug Administration’s (FDA) Expanded Access for Medical Devices Emergency Use mechanism (EAEU)

Several case reports and review articles have demonstrated success with NPWT in decreasing bacterial burden and stabilizing wounds in patients with NF, even within the head and neck regions [11–14]. Unfortunately, given the proximity of the wound bed to the lower eyelid and conjunctiva and surgical absence of the majority of the soft tissue of the eyelid including the orbicularis oculi, negative pressure wound therapy was contraindicated due to risk of traction injury to the globe and/or eyelid. To address this problem, a 3D-printed, patient-specific wound splint was designed at the point-of-care using the patient’s anatomy to facilitate NPWT. It was determined that the patient’s circumstances met the criteria for the FDA Expanded Access for Medical Devices Emergency Use mechanism (EAEU) with concurrence from the Institutional Review Board (IRB) (Fig. 2). A follow-up report was sent to the FDA within 5 days on the use of the device as required.

### Splint fabrication

**Fig. 3:**
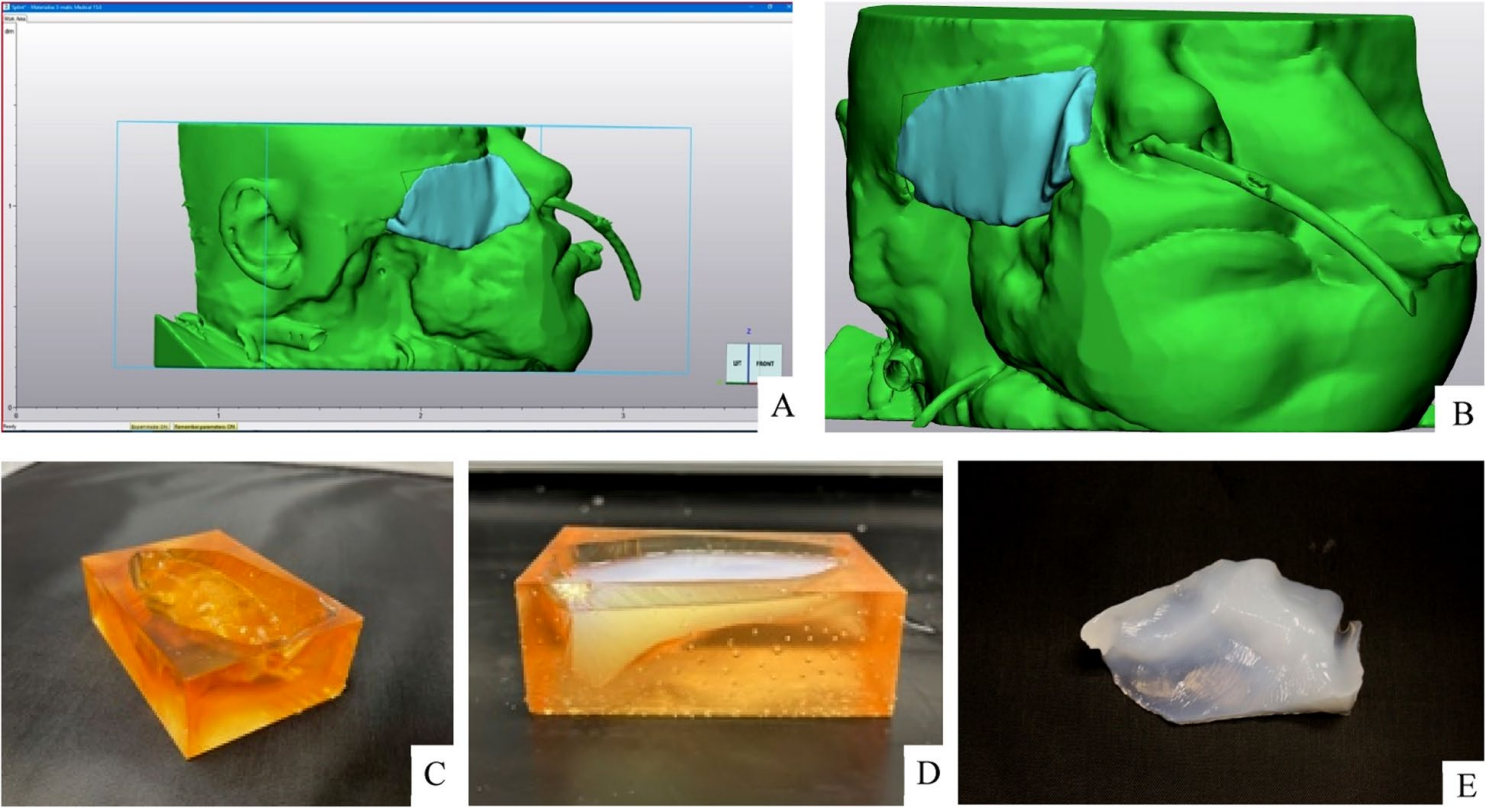
**a**, **b**) Design of wound splint based on patient computed tomography (CT) scan **c**) 3-dimensionally printed device mold **d**) Pouring of medical grade silicone into mold e) custom wound splint

A facial CT was obtained and Digital Imaging and Communications in Medicine (DICOM) images were uploaded to Materialise Mimics (Materalise, Leuven, Belgium). These were segmented to include the remaining soft tissue and bony structures in the nasofacial sulcus, periorbital, and malar regions. The native anatomy was then imported into Materalise 3-Matic (Materalise, Leuven, Belgium) and a 3-Dimensional (3D) surface was formed through the offset of the soft tissue and bony structures within the wound and extruded to provide additional surface and contact area. This process was performed with both clinician and engineering input to ensure correct design and adequate contact area for both the wound-vac device as well as the mount sutures. The resulting model was then smoothed with additional surface post processing to ensure fitment would be exactly along the contour of the wound and as well as provide a wide and flat surface on the outside upon which the NPWT could be secured (Fig. 3a&b). An open-faced mold was then designed in 3-matic to allow for a naturally smooth surface for the NPWT film to attach. The mold was printed on a FormLabs Form 3B in FormLabs Biomed Amber resin (FormLabs, Somerville, MA, USA) and post-processed following the recommended Form Wash and Cure time and temperatures for clinical applications (Fig. 3c). Factor II VST-50HD (FactorII, Inc., Lakeside, AZ, USA) platinum cure two part medical grade silicone was selected for its shore hardness of 38A and safe skin contact properties and was mixed in ratio of 10:1 A:B, degassed, and gravity poured into the mold (Fig. 3d). The mold and poured silicone were allowed to cure at 60 °C for 3 hours and the splint was extracted from the mold. The splint alone underwent an additional cure in at 150 °C for 2 h to ensure complete cure. The wound splint was then cleansed in 99% IPA for 5 minutes, before being ready for patient application (Fig. 3e).

An EAEU was submitted to the FDA on the basis of a life threatening infection requiring prolonged intubation that was necessary due to heavy sedation requirements with dressing changes. Additional concerns of development of orbital involvement and vision loss were cited if the wound did not begin to rapidly heal, and failure of other treatment options (wet-to-dry dressing changes). Concurrence was obtained from our IRB.

**Fig. 4:**
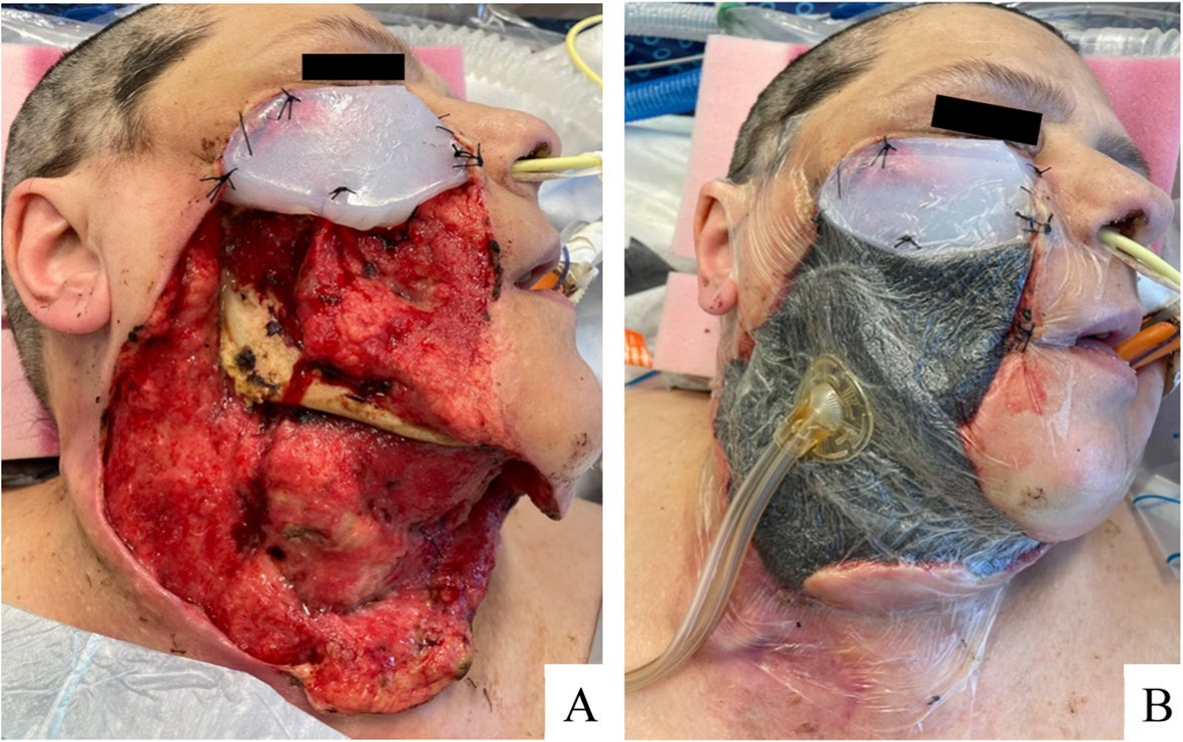
**a**) Placement of custom wound splint **b**) Successful securement of negative pressure wound therapy vacuum to wound splint

The wound splint was then placed at the superior edge of the wound bed, just below the remnant eyelid and sutured in place. A NPWT sponge was placed in the remaining wound bed. The NPWT film was applied directly to the splint, and ophthalmology confirmed that there was no traction on the lower lid (Fig. 4).

**Fig. 5:**
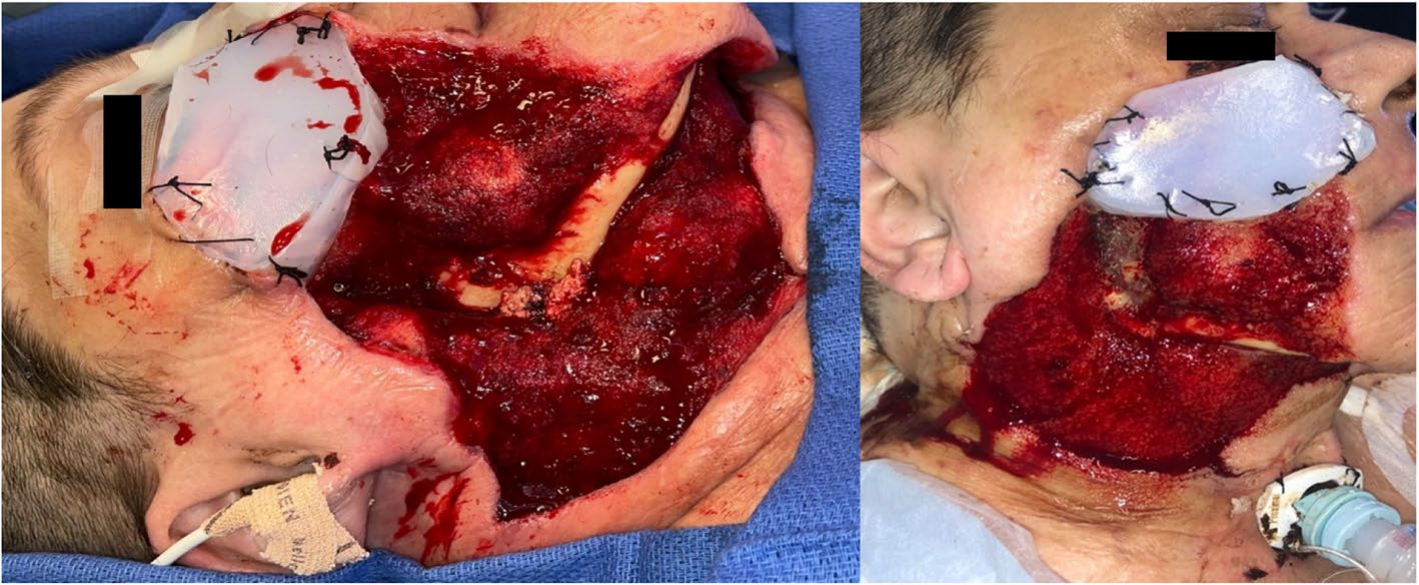
Healthy granulation tissue present in wound within 1 week of negative pressure wound therapy facilitated by splint

Since bedside dressing changes were no longer necessary, the patient was weaned from sedation, although she remained intubated. Two days following placement, the NPWT was exchanged in the operating room. The wound was noted to have stabilized, with well-healing granulation tissue present in > 50% of the wound bed, active bleeding throughout, and no evidence of devascularized areas or additional further purulence. The erythema of the mediastinal skin had improved, and the WBC count normalized. Five days later, the NPWT was again exchanged, and the dead space between the skin and trachea was noted to have healed and seated down, allowing for tracheostomy and removal of the endotracheal tube 2 weeks following initial placement (Fig. 5). Now fully weaned from sedation, the patient was transferred out of the intensive care unit and continued to express appreciation for her customized care and device.

**Fig. 6:**
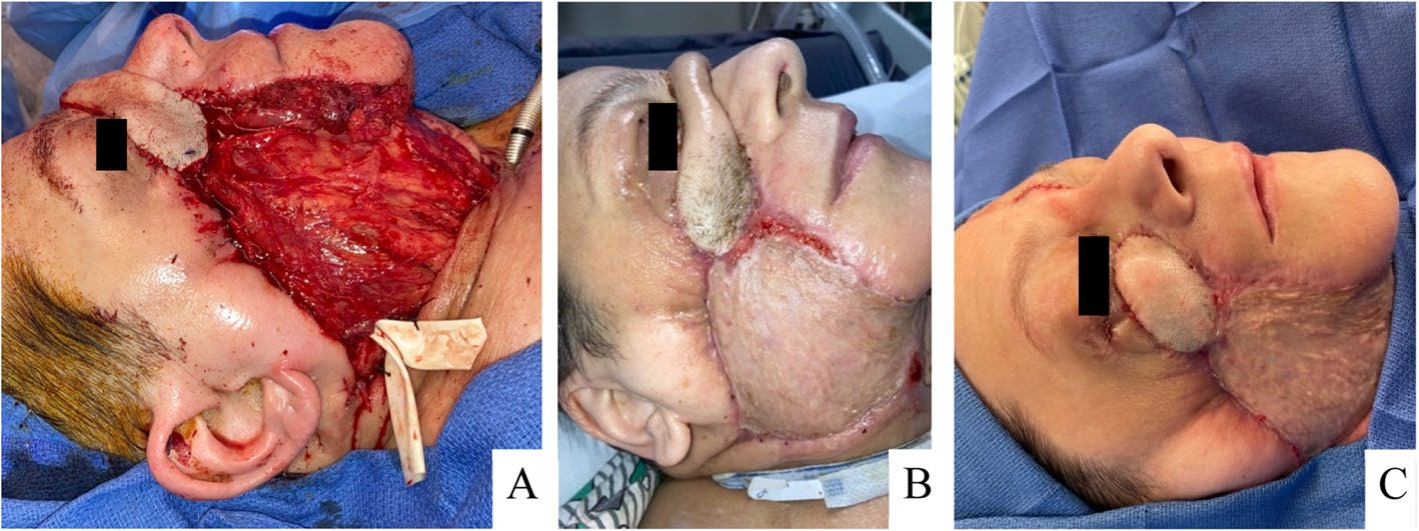
**a**, **b** Eventual reconstruction of hemifacial defect with paramedian forehead flap and right pectoralis major flap **c**) Results of second stage surgical reconstruction

With each exchange of NPWT, the wound continued to demonstrate contraction and healthy granulation tissue formation, with no evidence of ongoing infection. Four weeks after initial placement, the NPWT was removed, and the defect was reconstructed with a pectoralis muscle flap, split thickness skin graft, and right paramedian forehead flap (Fig. 6a,b).

A second stage surgery for division and inset was performed 4 weeks after the initial reconstruction (Fig. 6c). Following completion of reconstruction, vision testing and patient-reported vision were at baseline from prior to infection, with minimal lagophthalmos, no exposure keratopathy and no damage to the globe.

## Discussion

In this case, we present a situation in which 3D printing was used to design a patient specific device at the point-of-care, and placed under the FDA’s EAEU, in order to address a complex hemifacial wound from NF. The surgical management of NF involves early, aggressive debridement. The principles of wound management are to establish a healthy, uninfected wound bed for eventual reconstruction [1]. In facial NF, the proximity to vital structures may lead some to proceed with more cautious excision, but this can lead to persistence of disease, requiring more aggressive debridements in the future [15]. Even in the setting of aggressive initial debridement to healthy bleeding tissue, head and neck NF usually involves repeated debridements and a long course of local wound care prior to reconstruction.

In head and neck NF, previous case series have reported success with both frequent wet-to-dry dressing changes [3–5] or NPWT [6–9]. NPWT is thought to promote wound healing by removing secretions from the wound, decreasing the nidus for infection, reducing bacterial burden, and promoting vascularization of the wound bed and the formation of healthy granulation tissue [16]. While data comparing the use of standard wound care to NPWT are limited in the setting of acute infection, randomized evidence suggests accelerated wound healing time with the use of NPWT in other parts of the body [10].

In head and neck defects from massive infection and subsequent debridements, outcomes related to management of the wound bed are limited to case series. The original case series of this rare disease entity reported management of all types of wounds from NF with wet-to-dry dressing changes [3–5, 15]. NPWT has only recently been applied for management of NF in the head and neck [6–9]. These series found acceptable wound healing outcomes without recurrent or worsening infection. One group reported less aggressive debridement, keeping regions with discoloration or edema intact unless frankly avascular or necrotic [9]. All wounds eventually healed adequately for reconstruction, however, all patients required at least one additional debridement. In different series where standard debridement protocols were used and NPWT was applied, rates of repeated debridements were approximately 20% lower [6, 7].

NPWT is therefore a reasonable alternative to standard wound care in managing head and neck NF, particularly in the case of a large wound with suboptimal healing despite aggressive standard wound care, as presented in this case. Despite the potential benefits of NPWT, massive wounds in the HN present several unique difficulties to their application, which have previously been managed with various solutions [2, 7, 10, 13]. Particular challenges in this case include the proximity to the standard tracheostomy location and periorbita and risk of traction injury to the globe.

First, the proximity to a standard tracheostomy location in cases of cervical NF presents a challenge for long-term airway management. This is particularly relevant in the setting of NF of the HN as many present following Ludwig’s angina where airway management is an utmost concern [17]. Additionally, many patients with NF will require long-term ventilator support due to dissemination of and complications from infection, in which a tracheostomy may be indicated [5, 18]. In the presented case, the cervical portion of the wound extended near the midline and continued to expand despite aggressive local wound care. Placing tracheostomy through this area would have risked prolonged infection of the wound bed from tracheal secretions. Following NPWT, the wound decreased in size, allowing for safe tracheostomy placement without communication with the wound. Literature regarding NPWT in proximity to tracheostomy are limited. Balci et al. (2018) reported placement of foam to isolate the tracheostomy site from the wound bed [9]. Alternatively, a paramedian incision for tracheostomy has been reported in the case of a neck abscess [19], but these approaches are not well established. Therefore, delaying tracheostomy until the wound has decreased in size, which may be assisted by the use of NPWT, or been successfully reconstructed is reasonable and was pursued in this case.

Involvement of the orbit and periorbita with necrotizing fasciitis has previously been reported as a complication of pre-septal cellulitis [20, 21] or in the setting of facial necrotizing fasciitis [15]. Previous applications of NPWT to the periorbital region have been applied when the defect was superficial to the orbicularis oculi or in cases in which vision was already lost [15, 20]. Following NPWT for superficial periorbital infections, traction injury, vision loss, and permanent ectropion have not been reported [11, 20]. In contrast, in cases where facial NF extended to the deep periorbital tissue, debridement and local wound care have often resulted in permanent ectropion [15, 20]. To our knowledge, NPWT has not previously been applied to a periorbital wound bed deep to the orbicularis oculi in a patient with presumed intact visual function. The risk of potential eye injury related to the wound splint and NPWT use was weighed against the impending risk of infectious spread to the orbital compartment leading to vision loss, as previously reported. In this case, we successfully manufactured a patient-specific, 3D-printed wound splint separating the wound bed from the periorbita and allowing for safe application of NPWT. There was no obvious traction on the eye and the wound bed healed appropriately with preservation of vision and minimal ectropion due to wound contracture after reconstruction, and no wound splint related ocular complications. This concept has utility in relation to any vital or delicate structure, not just the periocular area.

### Expanded access for medical devices emergency use mechanism (EAEU)

In acute, life-threatening scenarios such as NF, emergency devices may be used through the EAEU mechanism of the United States FDA [22, 23]. Under the EAEU mechanism, the clinicians also certify there is no acceptable treatment alternatives and, due to the life-threatening nature of the scenario, there is no time to use existing pathways for FDA approval. After a physician determines that the scenario meets the EAEU criteria and has assessed potential benefits and risks of unapproved device use, as many patient protections as possible should be followed (Fig. 2).

Importantly, the EAEU must be reported to the FDA within 5 days of device use, and thus does NOT require prior authorization. The report must include a description of the device, explanation of management options that were previously attempted and exhausted (Fig. 2), the patient protections that were followed, and any available patient outcome information. This pathway remains the most expedited option available for physicians to treat patients with an acute, extraordinary condition [24].

There is little precedent in the literature regarding 3D printed, point of care medical devices in head and neck surgery. One of the most well-known applications of the FDA’s EAEU pathway within Otolaryngology was reported by Zopf et al. (2013) [25]. An externally placed 3D printed splint was secured to a patient’s trachea who had failed all other management options for severe tracheobronchomalacia. Ultimately, this point-of-care manufactured device served as an excellent temporary solution for a condition that the patient ultimately outgrew and otherwise would not have survived.

In this case, the patient had begun to develop severe ectropion and keratitis of her right eye, which ultimately posed a threat to her vision. Furthermore, she was at risk of respiratory suppression from pain medication boluses due to the extent of the daily dressing changes, which required her to remain endotracheally intubated on mechanical ventilation for over a month. Due to involvement of the visceral space in the infection, a tracheostomy was not possible, which required a prolonged intubation and threatened development of subglottic stenosis and tracheal injury as well as ventilator associated pneumonia. The above justification was presented to the IRB for concurrence and included in the FDA EAEU follow-up report, leading to the successful application of a custom, 3D printed silicone wound splint and favorable clinical outcome for the patient.

### Point of care device development

Point-of-care, patient-specific device manufacturing, particularly 3D modeling and printing, allows for better device customization, and lower time to treatment with potential for rapid production [26, 27]. In-house manufacturing of personalized medical devices also allows for more adjustments to each device in rapidly changing clinical scenarios such as NF. In contrast, commercial producers have limited ability to adapt to patient-specific scenarios and require a longer time (weeks) for model creation and delivery, supporting the effectiveness of an in-house protocol [28].

Macielak et al. (2020) also report the use of two 3D printed custom silicone fistula plugs for wound stabilization in a multi-recurrent laryngeal cancer who developed post-operative wound dehiscence and pharyngocutaneous fistulae [29]. In this case, point of care 3D printed devices also led to wound stabilization and ultimately allowed for safe reconstruction. These reports along with our application of a patient-specific 3D-printed device points to the therapeutic potential of such devices in the future. Currently, use of 3D printing in otolaryngology-head and neck surgery is limited to surgical planning while therapeutic applications are rare [30, 31]. Given the complexity of head and neck defects and the increased availability of and familiarity with 3D printing technology among high-volume otolaryngology centers, 3D printed devices should be considered in assistance of management of complex defects of the head and neck. Furthermore, this example demonstrates the pathway to create custom devices for unprecedented patient situations with no alternative therapies through the EAEU, opening up the possibility of point of care customization to all patients at high volume centers.

In conclusion, this case is the first reported use of a patient specific 3D printed wound splint to allow application of NPWT in close proximity to the orbit while preventing orbital injury and vision loss. This case highlights the potential for point-of-care medical devices to be utilized in complex soft tissue defects, particularly in the head and neck where wounds in close proximity to vital structures is a common occurrence. Additionally, this case highlights the successful use of the FDA’s EAEU pathway to manage a rapidly changing, life-threatening clinical scenario where no existing therapy remained a viable option. Together, these tools represent a powerful option for clinicians faced with unprecedented medical condition and may provide significant benefit to patients when utilized appropriately.

## Data Availability

Not applicable.

## Abbreviations

NF: Necrotizing fasciitis
NPWT: Negative pressure wound therapy
Wound vac: wound vacuum
3D: 3-dimensional
FDA: Food and Drug Administration
EAEU: Expanded Access for Medical Devices Emergency Use mechanism (EAEU)
IRB: Institutional review board
DICOM: Digital Imaging and Communications in Medicine
